# The Perspectives of the General Population and Relatives of Cancer Patients with Respect to the Do-Not-Resuscitate Order

**DOI:** 10.7759/cureus.3968

**Published:** 2019-01-26

**Authors:** Ahmed H Kaneetah, Feras O Baowaidan, Bahaa A Abulaban, Mahmoud F Sabban, Ahmad S Alshehri

**Affiliations:** 1 Internal Medicine, King Abdullah International Medical Research Center - King Saud Bin Abdulaziz University for Health Sciences, Jedah, SAU; 2 Internal Medicine, King Abdullah International Medical Research Center - King Saud Bin Abdulaziz University for Health Sciences, Jeddah, SAU; 3 Internal Medcine, King Abdullah International Medical Research Center - King Saud Bin Abdulaziz University for Health Sciences, Jeddah, SAU; 4 Oncology, King Abdullah International Medical Research Center - King Saud Bin Abdulaziz University for Health Sciences, Jeddah, SAU

**Keywords:** do not resuscitate order, dnr, saudi family, knowledge, acceptance

## Abstract

Background

A do-not-resuscitate (DNR) order is a medical decision that instructs healthcare providers to withhold cardiopulmonary resuscitations (CPR) to patients in case of cardiopulmonary arrest in respect to their wishes. In Saudi Arabia, the decision is usually made by physicians based on the Fatwa number 12086 regardless of the patients’ or their families' desires. Assessing the knowledge, perception, and attitude of Saudi family members towards this medical decision may help guide medical practitioners to make decisions that are legally and ethically acceptable for the patients and their family. Therefore, this study aimed to assess their knowledge, perception, and attitude about DNR decisions and to determine demographic variables that affect their attitude towards DNR decisions.

Method

This cross-sectional study was conducted from December 2017 to January 2018 utilizing survey distribution through emails and different social media outlets including Facebook, Twitter, Pinterest, and LinkedIn. A self-administered questionnaire was employed to elicit responses regarding knowledge, perception, and attitude towards DNR decisions. Statistical Package for the Social Sciences Windows version 17 (SPSS v.17) (IBM Corporation, USA) was used for data management and analysis.

Result

Of the 1882 participants who filled the questionnaire, only 1693 participants were eligible as the study sample population and were included in the final data analysis. Most of the participants were from the Makkah region (72.2%) and were mainly from the general population (61.66%). Participants were mainly females (66%) and within the median age of 30 years (IQR: 21). As expected, participants who had heard about the DNR practice were from the medical field (58.6%) and they were mainly distant relatives. Almost 76% of the participants had two to four incorrect answers about the DNR practice, and it indicated that participants have inadequate knowledge about a DNR order. Most of the participants (43.2%) refused to be on a DNR order if they were diagnosed with a terminal illness. However, most participants (69.9%) wanted to have an involvement in the decision-making of being on a DNR order. In terms of willingness to understand and learn about the DNR decision, 1475 (87.1%) of the participants wanted to learn more about the DNR practice. Being a relative of a terminally ill cancer patient did not have a significant effect on the knowledge and perceptions of participants about being on a DNR order. However, having a background in medicine was found to be significantly associated with their acceptance to be on a DNR order. The knowledge score regarding DNR was found to be significantly associated with higher acceptance towards DNR orders.

Conclusion

The majority of participants had a lack of knowledge about the DNR practice. Their religion’s concept of hope and virtue is considered as the major reason for their DNR order refusal. However, proper education about the DNR practice and involvement in the DNR order decision-making will increase the participants’ knowledge and will improve their acceptance of the DNR practice.

## Introduction

A do-not-resuscitate (DNR) order is a medical decision that instructs healthcare providers to withhold cardiopulmonary resuscitations (CPR) to patients in case of cardiopulmonary arrest in respect to their wishes. It is usually suggested by physicians to terminally ill patients. However, in Saudi Arabia, the decision is usually made by physicians regardless of the patients’ or their families' desires. This is according to the Fatwa number 12086 by the General Presidency of Scholarly Research and Ifta, which is an acceptable source of legislation. It also states that: “If three knowledgeable and trustworthy physicians agree that it is not appropriate to resuscitate a patient, when it is evident that they are suffering from an obstinate, incurable illness and that death is inevitable, there is no need to use resuscitators, life-supporting machines” [1].

Multiple studies in the literature have shown that demographic variables such as age, race, religion, marital status, and educational level are associated with different knowledge, perception, and attitudes towards DNR orders [2-6]. Thus, a local study is needed to assess the knowledge, perception, and attitudes towards DNR orders in Saudi Arabia.

DNR orders are often questioned on ethical and religious grounds because 1) it appears as if physicians are letting their patients die, and 2) even if they resuscitate the patient and let the patient survive, the resuscitation will not cure the disease; this also sometimes leads to more complications that prolong the patients’ suffering. According to the American Heart Association (AHA) report in 2016, the success rate of CPR in the general population despite their medical illness is 24.8% inside hospitals and much less outside [7]. According to a systematic review, the chance of survival to discharge after CPR for advanced cancer patients is 10.1% if the resuscitation occurs in the wards, and 2.2% if it occurs in intensive care units (ICUs) [8]. Moreover, the return of spontaneous circulation (ROSC) is lower in patients with cancer [9] and most of those with advanced cancer in whom CPR was successful die within days to weeks in the ICU [10-13].

Previous studies in Saudi Arabia have evaluated knowledge and attitude of outpatients, interns, and residents towards DNR orders [14-16]. However, there is no study that assessed the knowledge, perception, and attitudes towards DNR orders among the general population as well as cancer patients' relatives in Saudi Arabia. In addition, there are no studies in Saudi Arabia that evaluated how demographic variations can affect the knowledge, perception, and attitudes towards DNR orders.

Relatives of cancer patients are the ones who usually witness and experience the DNR practices in hospitals. Assessing their knowledge, perception, and attitude towards this medical decision may help guide medical practitioners to make decisions that are legally and ethically acceptable for the patients and their families. Therefore, this study aimed to assess their knowledge, perception, and attitudes about DNR decisions and to determine demographic variables that affect attitudes towards DNR decisions.

## Materials and methods

This cross-sectional study was conducted from December 2017 to January 2018. It aimed to determine the perspectives of the general population and relatives of cancer patients with respect to the DNR order. Respondents were recruited nationally and internationally through invitations in emails and different social media outlets including Facebook, Twitter, Pinterest, and LinkedIn. Eligible Saudi respondents were those aged 18 years old and above, as well as who had experienced or witnessed a family relative diagnosed with terminal cancer. Non-Saudi and those have been diagnosed with cancer were excluded.

A self-administered questionnaire developed by the authors was employed. The face and construct validity of the questionnaire was addressed through consultation with three oncology and two intensive care consultants, as well as with several family members and relatives of the terminally lll cancer patients in Princess Noorah Oncology Center, King Abdulaziz Medical City, Jeddah, Saudi Arabia. Individual items were evaluated based on their relevance to DNR content domain. Items were also evaluated based on their affective and cognitive representativeness to the target population of this study. Thus, the final questionnaire developed consisted of three parts. The first part dealt with the demographical data of the respondents, which included questions regarding their age, gender, marital status, region, income, educational level, employment, and medical background (i.e. student or work related to medicine). The respondents were also asked if they had family members who had ever been diagnosed with cancer and whether they had heard about the DNR practice before. The second part consisted of seven multiple-choice questions that assessed the respondents’ knowledge regarding the concept of DNR. The third part consisted of five questions that assessed the attitude of respondents towards DNR decisions. It included questions that asked the respondents about their willingness to be on a DNR order if they were diagnosed with a terminal illness and if they wished to be involved in the DNR decision-making. It also included questions to assess the respondents’ willingness to discuss DNR decisions and learn more about it.

All data collected were grouped according to the degree of family relationship with regard to the terminally ill cancer patient. First-degree relatives included parents, siblings, children, and spouses. Second-degree relatives included grandparents, grandchildren, uncles, aunts, first cousins, nephews, and nieces. Distant relatives included relatives who are not first- or second-degree relatives. General populations were those who did not have any relation to the cancer patient.

Statistical Package for Social Sciences Windows version 17 (SPSS v.17) (IBM Corporation, USA) was used for data management and analysis. Descriptive analysis, standard deviations, and percentages were performed for normally distributed variables, while mean and interquartile range (IQ) were used for skewed distributed variables. To determine significant associations between categorical variables, the chi square test was used. The Kruskal-Wallis test was used to test for differences in the knowledge among the different subgroups regarding the DNR practice. Cross tabulation was done to compare attitudes towards the DNR decisions among the different subgroups. Lastly, Pearson’s regression analysis was employed to determine confounding factors. Statistical significance was set at p < 0.05.

## Results

Demographic data

Of the total 2830 Saudi survey participants invited, 1882 (i.e. 66.50% response rate) of them filled the questionnaire. However, those who did not meet the eligibility criteria and those who did not completely fill the questionnaire were excluded. Thus, only a total of 1693 respondents were included in the final analysis.

Analysis showed that most of the respondents were females (66%) and mainly within the median age of 30 years (IQR: 21). Respondents were from the 13 administrative regions of Saudi Arabia and most of them were from the Makkah region (72.2%). Respondents mostly had a bachelor’s degree (51.1%) and were in non-medical fields (67.6%).

The study sample population comprised of 281 (16.5%) first-degree relatives of the cancer patient, 327 (19.3%) were second-degree relatives, 33 (1.9%) were distant relatives, and 1052 (61.66%) were from the general population. The complete details of the respondents’ demographics for each group are presented in Table 1.

**Table 1:** Participants' demographic characteristics.

Demographic Characteristics			All	Degree of Family Relationship			
				1^st^ Degree	2^nd^ Degree	Distant Relative	General Population
			Median (IQR)	Median (IQR)	Median (IQR)	Median (IQR)	Median (IQR)
Age			30 (21)	42 (20)	25 (17)	23 (13)	29 (18)
			n (%)	n (%)	n (%)	n (%)	n (%)
Gender		Male	564 (33.3)	90 (32)	77 (23.5)	10 (30.3)	387 (36.8)
		Female	1129 (66.7)	191 (68)	250 (76.5)	23 (69.7)	656 (63.2)
Marital status		Married	871 (51.5)	188 (66.9)	141 (43.1)	11 (33.3)	531 (50.5)
		Not married	821 (48.5)	93 (33.1)	186 (56.9)	22 (66.7)	520 (49.5)
Region		Makkah	1218 (72.2)	209 (74.4)	234 (72.2)	27 (81.8)	748 (71.3)
		Riyadh	161 (9.5)	21 (7.5)	30 (9.3)	1 (3)	109 (10.4)
		Eastern province	129 (7.6)	26 (9.3)	36 (11.1)	4 (12.1)	63 (6)
		Madinah	52 (3.1)	9 (3.2)	8 (2.5)	00	35 (3.3)
		Asir	43 (2.5)	5 (1.8)	5 (1.5)	00	33 (3.1)
		Other regions	72 (4.3)	9 (3.2)	10 (3.1)	1 (3)	52 (5)
		Outside	12 (.7)	2 (.7)	1 (.3)	00	9 (.9)
Income		≤10000	714 (42.2)	111 (39.5)	135 (41.3)	12 (36.4)	456 (43.3)
		>10000	979 (57.8)	170 (60.5)	192 (58.7)	21 (63.6)	596 (56.7)
Educational level			28 (1.7)	8 (2.9)	3 (.9)	00	17 (1.6)
		High school	486 (28.7)	47 (16.8)	119 (36.4)	17 (51.5)	303 (28.8)
		Diploma	156 (9.2)	27 (9.6)	24 (7.3)	1 (3)	104 (9.9)
		Bachelor	864 (51.1)	155 (55.4)	153 (46.8)	12 (36.4)	544 (51.7)
		Higher education	158 (9.3)	43 (15.4)	28 (8.6)	3 (9.1)	84 (8)
Employment		Employed	733 (43.4)	141 (50.5)	119 (36.4)	10 (30.3)	463 (44.1)
		Not employed	237 (14)	38 (13.6)	51 (15.6)	3 (9.1)	145 (13.8)
		Student/trainee	569 (33.7)	42 (15.1)	136 (41.6)	18 (54.5)	373 (35.5)
		Retire	151 (8.9)	58 (20.8)	21 (6.4)	2 (6.1)	70 (6.7)
Specialty		Medical	548 (32.4)	64 (22.8)	133 (40.7)	10 (30.3)	341 (32.4)
		Non-medical	1145 (67.6)	217 (77.2)	194 (59.3)	23 (69.7)	711 (67.6)
		Total	1693	281	327	33	1052

Knowledge about DNR

We found that 541 (32%) respondents had heard about the DNR order before. As expected, most of them were from the medical field (58.6%) as compared to a non-medical field (19.2%). The majority were distant relatives (45.5%) followed by second-degree relatives (38.8%), general population (30.5%), and first-degree relatives (27.8%). The items that assessed DNR knowledge found that majority (54%) of the respondents answered four or five questions correctly, 25.4% answered six or seven questions correctly, and 20.6% answered three questions or fewer correctly. Almost 76% of the respondents had two to four incorrect answers about the DNR practice. The median knowledge score was five out of seven. However, Table 2 shows that having a relative with cancer doesn’t have an influence on the DNR knowledge score (p=0.649). The only demographic variable that was associated with better knowledge score is being in a medical field (p=0.003) (Table 3).

**Table 2:** Knowledge score when grouped according to degree of family relationships.

Degree of Family Relationships		n (%)	Kruskal-Wallis P value
General		1052 (61.66)	0.649
Relatives	1^st^	281 (16.5)	
	2^nd^	327 (19.3)	
	3^rd^	33 (1.9)	

**Table 3:** Multivariate regression analysis of demographic variables in association with knowledge.

Demographic Variables	Subgroups	Adjusted P value
Age	Median (IQR) 30 (21)	0.67
Gender	Male	0.971
	Female	
Marital status	Married	0.78
	Not married	
Income	≤10000	0.68
	>10000	
Educational level		0.77
	High school	
	Diploma	
	Bachelor	
	Higher education	
Employment	Employed	0.64
	Not employed	
	Student/trainee	
	Retire	
Specialty	Medical	0.003
	Non-medical	

Attitude towards DNR

Most of the respondents (43.2%) refused to be on a DNR order if they were diagnosed with a terminal illness. The most common reasons of refusal were of hope and religious concern. Other reasons were related to their responsibilities and family-related concerns.

Most respondents (69.9%) wanted to be involved in the decision-making of being on a DNR order. Considering life situation or circumstances, there were 1229 (72.6 %) respondents who wanted to discuss DNR orders while they were in good health, 269 (15.9 %) wanted to discuss it only if they were diagnosed with a terminal illness, and 195 (11.5 %) did not want to discuss it at all. In terms of willingness to understand and learn about DNR decisions, there were 1475 (87.1%) respondents who wanted to learn more about the DNR practice. Being a cancer patient's relative did not affect their preference to be on a DNR order (Table 4). Furthermore, demographic variations were found not associated with any change in acceptance of being on a DNR order except for being in a medical field. Being in a medical field is associated with higher acceptance of being on a DNR order (p=0.002) (Table 5).

**Table 4:** Acceptance towards DNR when grouped according to degree of relatives.

		If I were diagnosed with a terminal illness, I prefer to be on DNR.			P Value
		Agree (%)	Neutral (%)	Disagree (%)	
General		294 (27.9)	298 (28.3)	460 (43.7)	0.27
Relatives	1^st^ degree	93 (33.1)	71 (25.3)	117 (41.6)	
	2^nd^ degree	108 (33)	82 (25.1)	137 (41.9)	
	Distant	6 (18.2)	9 (27.3)	18 (54.5)	
All		501 (29.6)	460 (27.2)	732 (43.2)	

**Table 5:** Acceptance towards DNR between medical and non-medical respondents.

		If I were diagnosed with a terminal illness, I prefer to be on DNR			
		Agree (%)	Neutral (%)	Disagree (%)	sig
Specialty	Medical	186 (33.9)	158 (28.8)	204 (37.2)	0.002
	Non- medical	315 (27.5)	302 (26.4)	528 (46.1)	

Association between knowledge and attitude towards DNR

Lastly, this study found that knowledge score is significantly associated with high acceptance towards the DNR order (p=0.007) (Table 6).

**Table 6:** Association between knowledge and acceptance towards DNR.

	n (%)	Mean Rank	Kruskal-Wallis P value
Agree	501 (29.6)	888.69	0.007
Neutral	460 (27.2)	865.95	
Disagree	732 (43.2)	806.56	

## Discussion

The DNR decision of patients is legal in some countries and prohibited or restricted in some other countries. In addition, implementation of DNR orders is a controversial issue. Do we harm patients with DNR? Some studies have presented the effects of DNR on the ROSC and results of CPR among patients with malignancy and end-stage disease [9,17] and therefore consider the DNR order as an acceptable option for patients with terminal cancer. On the other hand, DNR orders are usually questioned because they can be perceived as antireligious or ethically unacceptable. Saudi Arabia is one of the most religious countries in the world and the distinct Saudi culture also has different ethical and moral standards compared to those of other parts of the world. These differences have a great impact on the knowledge, perception, and attitude towards very sensitive topics such as DNR. In our study 32% of all respondents have heard about the DNR practice before. This is much less than another study done in outpatient clinics in Riyadh, Saudi Arabia in 2017 and a Canadian study done in primary care centers in North Vancouver in 2009. The percentage of those who have heard about the term DNR before were 75% and 84%, respectively [4, 16]. Contrary to what we hypothesized, there is no association between the relationship with the patient and having heard about DNR before. There is no study in the related literature that investigates this association. As expected, those who study or work in the medical field have heard more about DNR orders (58.6%) in comparison to those who did not study or work in the medical field (19.2%). One study done among interns and residents in Jeddah, Saudi Arabia in 2016 showed that 92% of residents and 70% of interns were familiar with the term DNR [14]. In another study in Hong Kong in 2007, 66.4% of non-medical students and 18.7% of medical students had never heard of DNR [18]. What could explain the lower percentages in our study is that we included all those who study or work in any medical field that might not be exposed to DNR orders.

Having a relative with cancer was not associated with better knowledge about DNR. This can be explained by the fact that DNR is a unilateral decision in Saudi Arabia. Therefore, physicians do not explain the DNR practice properly to their patients and the relatives, which might lead to insufficient hospital education. Only a single study in Canada with nine respondents in a care givers’ group has assessed the understanding regarding the term DNR, and six out of the nine respondents showed good knowledge [19].

In a study in the United States among inpatients admitted for general medical services, 16% refused to be on DNR if they were diagnosed with a terminal illness in comparison to 43.2% of the respondents [5]. The most common reasons for refusal in the said study were hope, religious reasons, or both. It is also worth noting that although the decision in Saudi Arabia is solely made by physicians, most of the respondents wanted to be involved in the decision-making.

According to a study done in Saudi Arabia among outpatients, 90% of the respondents wanted to discuss DNR while they were ill and 10% while they were healthy [16]. In another study in Canada, 56% of the respondents preferred to discuss the matter when they were still healthy [4]. Interestingly, 72.6% of the respondents wanted to discuss DNR orders while they are in good health, 15.9% wanted to discuss it only if they are diagnosed with a terminal illness, and 11.5% did not want to discuss it at all. Having a relative with cancer does not play a significant role in the preference of being on a DNR order. As expected, having more knowledge is associated significantly with higher acceptance. In addition, most respondents wanted to learn more about DNR orders. Consequently, more education is needed in the hospital setting regarding this matter as most are keen to learn more about this topic, which can improve the acceptance rate. In addition, physicians might consider discussing these topics with their patients when they are still healthy.

## Conclusions

The majority of participants had a lack of knowledge about the DNR practice. The concept of hope and virtue of their religion were considered as the major reasons for DNR refusal. However, being in a medical field is a significant factor in understanding the concept of DNR acceptance. The study only goes to show that educating family members and relatives about DNR and involving them in the DNR decision-making is essential to improve their knowledge and acceptance of DNR orders.
